# Combined Heat and Mass Transfer Associated with Kinetics Models for Analyzing Convective Stepwise Drying of Carrot Cubes

**DOI:** 10.3390/foods11244045

**Published:** 2022-12-14

**Authors:** Prarin Chupawa, Wanwisa Suksamran, Donludee Jaisut, Frederik Ronsse, Wasan Duangkhamchan

**Affiliations:** 1 Research Unit of Mechatronics Engineering, Faculty of Engineering, Mahasarakham University, Kamriang, Kantarawichai, Maha Sarakham 44150, Thailand; 2 Research Unit of Smart Process Design and Automation, Mahasarakham University, Kamriang, Kantarawichai, Maha Sarakham 44150, Thailand; 3 Department of Food Technology, Faculty of Technology, Mahasarakham University, Kamriang, Kantarawichai, Maha Sarakham 44150, Thailand; 4 Department of Farm Mechanics, Faculty of Agriculture, Kasetsart University, Lard Yao, Chatuchak, Bangkok 10900, Thailand; 5 Department of Green Chemistry and Technology, Faculty of Bioscience Engineering, Ghent University, Coupure Links 653, B-9000 Ghent, Belgium; 6 Research Unit of Process Design and Automation, Faculty of Engineering, Mahasarakham University, Kamriang, Kantarawichai, Maha Sarakham 44150, Thailand

**Keywords:** finite element, kinetics modeling, optimization, drying simulation

## Abstract

Stepwise drying is an effective technique that promotes energy saving without additional capital cost. The stepwise drying mode was investigated for energy consumption and dried product qualities using a coupled heat and mass transfer model associated with kinetics equations of volume shrinkage and degradation of β-carotene in carrot cubes. Simulations were performed using a finite element method with extension of a chemical species transport. Validation experiments were carried out under constant drying modes at 60 °C, 70 °C and 80 °C using a lab-scale convective hot air dryer. The verified models were subsequently employed to investigate the effects of two step-up drying modes (60 to 70 °C and 60 to −80 °C). The optimal drying condition was determined using the synthetic evaluation index (SI) with criteria of high specific moisture evaporation rate (SMER), low shrinkage ratio and β-carotene degradation. Simulated results showed comparable agreement with experimental data of moisture content, shrinkage ratio and β-carotene ratio. Step-up drying of 60 to 70 °C gave the highest SMER of 0.50 × 10^−3^ kg of water evaporated per kWh, while the operation at constant temperature of 80 °C gave the lowest value of 0.19 × 10^−3^ kg of water evaporated per kWh. Model-predicted results showed less shrinkage of carrot cubes, but higher degradation of β-carotene under step-up drying compared to single-stage drying under temperature of 60 °C. Based on the highest SI value (0.36), carrot cubes were optimally dried under step-up mode of 60 to 70 °C.

## 1. Introduction

Carrots are a popular vegetable and have a long-chain polyene structure [1,2,3]. When newly harvested carrots contain high moisture content, this needs to be reduced to a safe level to inhibit the growth of microorganisms and prolong shelf life during storage [4]. Drying removes water from the wet materials by means of simultaneous heat and mass transfer mechanisms. Removal of water or other liquids from solid materials due to evaporation causes changes in dried product qualities [5,6]. Convective drying is most widely used for fruits and vegetables due to its simplicity and operational cost effectiveness [4].

During drying, the water mass transfer mechanism occurs in two stages involving a constant-rate period and a falling-rate period controlled by external and internal mass transfer, respectively [7,8]. In convective hot air drying for biological materials, the latter stage reflects extensive energy consumption because of the higher internal mass transfer resistance caused by the difficulty of water transportation through progressively thicker layers of dry matrix under constant heat supply [9,10]. Therefore, this period normally requires drying time with lower energy efficiency.

Intermittent stepwise drying is used to improve energy efficiency and product quality without increasing capital costs [11] by varying the drying air temperature [12]. During the falling-rate drying stage, moisture at the material core is removed by a constant thermal energy with low diffusion rate, resulting in decreasing drying rate and prolonged drying time. Shortening this period by enhancing the moisture diffusion rate reduces energy consumption [12,13]. Stepwise drying under faster diffusion speeds improves the process, consequently lowering energy consumption. Stepwise drying has been used as an alternative technique to improve energy efficiency but hot air provokes changes in physical and chemical properties [13]. Thus, understanding how the heat and mass transfer mechanisms affect physical and chemical properties should be taken into consideration for process design and optimization. 

Numerical models have been proposed as powerful tools to explore the transfer phenomena taking place during the drying process, compared with trial-and-error experiments [13,14]. In the drying process, complex heat and mass transfer equations are governed in partial differential form and solved numerically by numerical methods such as finite difference, finite volume and finite element scheme [14,15,16,17]. Among these, the finite element (FE) approach has been widely applied in the food drying process to elucidate the transport phenomena, providing high-accuracy solutions for complex geometries [16,18]. Curcio et al. [19] used the simplified finite element model to obtain insights into all transfer phenomena taking place during convective drying of cylindrical carrot samples. This FE model of practical drying behavior showed good agreement between predicted and experimental results but did not explain interfacial heat and mass transfer. To obtain more realistic results, an FE model was developed to solve a coupled set of partial differential equations to simulate the simultaneous heat and mass transfer mechanism occurring during the drying process of injera [20], carrot slices [21] and macadamia nuts [22]. Apart from temperature and moisture ratio, shrinkage of potato cylinders was also captured using the coupled heat and mass transfer associated with moving mesh modules based on the finite element approach [23]. The verified FE model effectively predicted the temperature increment and described drying characteristics of the samples with different sizes. To obtain more accurate simulated results, Yuan et al. [24] included shrinkage deformation in an FE model of the apple-slice drying process while Kumar et al. [25] solved equations of multiphase transport in apple drying using the finite element method. The FE model predicted moisture contents of apple slices as a function of drying time which agreed well with the experimental results. The verified model showed higher fluxes of liquid water and vapor close to the sample surface. Seyedabadi et al. [26] employed the validated FE model to optimize the drying process of cylindrical banana using both quality and efficiency criteria.

As described above, the FE model has been employed as a promising tool to explore the transfer phenomena taking place in the drying process of fruits and vegetables. However, solving the complex equations of all transport phenomena using the finite element approach associated with the coupled heat and mass transfer model is both difficult and time consuming. Simplified approaches such as empirical correlations could be another choice for understanding the heat and mass transfers in practical or industrial design to optimize the drying process [18,27]. Therefore, this work presents the feasibility of using a simple FE model associated with kinetics equations for evaluating stepwise drying performance and product qualities.

An extensive simulation of stepwise drying was presented by Jomlapelatikul et al. [28], using a coupled heat and mass transfer model in a framework of finite element to determine a suitable intermediate point for changing the stepwise drying temperature. The appropriate changing point was chosen with respect to both drying time and energy consumption. This study simulated the drying characteristics of carrot cubes under stepwise drying mode using a simplified heat and mass transfer model associated with the empirical correlations of volume shrinkage and degradation of β-carotene. The verified model was then used to describe the effect of stepwise drying modes on both physical and chemical properties in terms of volume shrinkage and degradation of β-carotene. The optimal drying condition was determined based on the drying performance and energy consumption and, also on the dried product quality attributes using the synthetic evaluation index.

## 2. Materials and Methods

### 2.1. Description of the Coupled Heat and Mass Transfer Model

During the drying process, heat is transferred simultaneously by convection and conduction from the drying medium to the product core. The material surface is first heated under convection from the air and heat is subsequently transferred toward the core by conduction. Water vapor due to evaporation simultaneously diffuses counter-currently toward the surface and is transferred to the drying medium [28,29]. Here, coupled heat and mass transfer in carrot cubes during the convective drying process were associated with both the kinetics of volume shrinkage and β-carotene degradation. The governing equations of heat and mass transfer were solved by the finite element method.

The effect of drying air flow was negligible, and constant heat flux was defined on the surface of the carrot cubes. Therefore, only heat conduction inside the cube was taken into account and described by

(1) $$\rho C_{P}\frac{\partial T}{\partial t}=\nabla \cdot \left(k\nabla T\right)$$

Carrot cube properties, following [28], used in the heat conduction model (Equation (1)), included density (*ρ*) of 1154 kg m^−3^ and specific heat capacity (*C_p_*) of 3736 J kg^−1^ K^−1^. The thermal conductivity, *k* (W m^−1^ K^−1^) is mainly affected by air temperature and was estimated by an empirical equation proposed by [30], with *T* expressed as absolute temperature (K).

(2) $$k=0.5464+0.0012T-2.0\times 10^{-6}T^{2}$$

Local moisture content during carrot sample drying was simulated based on Fick’s diffusion law [31], as expressed in Equation (3). The effective diffusivity, *D_eff_* (m^2^ s^−1^), in this equation was estimated using the empirical model proposed by Botelho et al. [31], as described in Equation (4), where *X* is moisture content expressed as dry basis and *T* is expressed in °C.

(3) $$\frac{\partial X}{\partial t}=\nabla \cdot \left(D_{eff}\nabla X\right)$$

(4) $$D_{eff}=exp\left[-0.97-\left(\frac{3459.8}{T+273}\right)+0.059X\right]/3600$$

The above diffusion equations used for simulating the moisture content were numerically solved using the initial conditions of uniform temperature (*T_0_*) and moisture content (*X_0_*) in a cartesian coordinate.

Equations (5) and (6) are boundary conditions assuming that loss of heat and moisture through the carrot cube surface was due to convection [28,29,31].

(5) $$-n\cdot k\nabla T=h_{T}\left(T_{a}-T\right)+H\cdot \left(D_{eff}\nabla X\right)$$

(6) $$-n\cdot \left(D_{eff}\nabla X\right)=k_{m}\left(X_{e}-X\right)$$

The correlations of enthalpy (*H*, J mol^−1^) proposed by Hii et al. [13] (expressed in Equation (7)), heat transfer coefficient (*h_T_*, W m^−2^ K^−1^) (expressed in Equation (8)) and mass transfer coefficient (*k_m_*, m s^−1^) proposed by Botelho et al. [31] (expressed in Equation (9)) were employed to solve the above heat and mass fluxes through the material surface.

(7) $$H=-0.0469T^{2}-13.493T+5220$$

(8) $$h_{T}=0.624T+16.7261$$

(9) $$k_{m}=0.001T+0.0043$$

In Equations (5)–(9), *T* denotes absolute temperature (K) and *T_a_* is the set-point drying air temperature (K). Moisture contents at a specific time and equilibrium, expressed as dry basis, are denoted by *X* and *X_e_*, respectively.

Following Jomlapelatikul et al. [28], the finite element approach was employed to estimate the heat transfer rate (*Q*, J s^−1^) under two step-up temperatures starting from ambient temperature of 25 °C to 60 °C for the first stage and 60 °C to 70 °C for the second stage, and from 25 °C to 60 °C for the first stage and 60 °C to 80 °C for the second stage. The heat transfer rate for both step-up drying modes was calculated by Equation (10), where m and Δ*T_a_* are air mass flow rate (kg s^−1^) and temperature difference between ambient and drying temperature, respectively.

(10) $$\dot{Q}=\dot{m}C_{P}\Delta T$$

It was assumed that energy was mainly utilized by the heating air system, with no heat loss in the drying system [13]. Therefore, the energy consumed by the air supply system and other electric devices was negligible when calculating total thermal energy. The thermal energy utilized in the step-up drying mode of the two drying stages was calculated using two heat transfer rates as ${\dot{Q}}_{1}$ for the first stage and ${\dot{Q}}_{2}$ for the second stage and expressed by Equation (11). Durations for the first stage and the second stage were *t_1_* and *t_2_* (min), respectively.

(11) $$Q={\dot{Q}}_{1}\Delta t_{1}+{\dot{Q}}_{2}\Delta t_{2}$$

Specific moisture evaporation rate (*SMER*), defined as the energy required to remove one kilogram of water, was used as an indicator to evaluate the drying performance; lower *SMER* indicated higher energy efficiency. The *SMER* was calculated as the ratio between water removal and total thermal energy [28], as follows:

(12) $$SMER=\frac{w_{i}-w_{f}}{Q}$$

where *w_i_* and *w_f_* are initial and final weight of the carrot cubes, respectively.

### 2.2. Kinetics Modeling

#### 2.2.1. Materials

Fresh carrots (*Daucus carota* L.), purchased from a local market in Maha Sarakham Province, Thailand, were washed, peeled and cut into 1 cm cubes. Initial moisture content was determined by the standard oven method. The sample was dried in a hot air oven under 103 °C for 72 h. All chemicals used for analyzing β-carotene content, including ethanol, *n*-hexane, methanol, acetonitrile and dichloromethane, and for measuring solid volume, including *n*-heptane were obtained from Merck (Darmstadt, Germany), while β-carotene standard was obtained from Sigma-Aldrich (St. Louis, MO, USA).

#### 2.2.2. Kinetics of Volume Shrinkage

Shrinkage, expressed by change in volume of carrot cubes during processing, was analyzed by fluid replacement (Archimedes’ principle) associated with *n*-heptane. Shrinkage percentage was calculated using the volume before (*V*_0_) and after drying (*V_t_*) at each time interval, as expressed in Equation (13).

(13) $$\%\mathrm{shrinkage}=\frac{V_{0}-V_{t}}{V_{t}}\times 100$$

The shrinkage data were fitted to several empirical models summarized by Koç et al. [32] with slight modification. The shrinkage percentage was correlated with moisture content at a specific drying time, as summarized in Table 1. The most suitable equation for describing the shrinkage kinetics was obtained using *R^2^*, *RMSE* and *χ^2^*.

#### 2.2.3. Degradation Kinetics of β-Carotene

Concentration of β-carotene was determined according to Jamali et al. [3]. Briefly, 3 g of dried carrot cubes were added to acetone solution and mixed using a vortex for 30 s. The mixture was then centrifuged at 2100× *g* for 5 min. The supernatant was filtered through Whatman No. 1, evaporated at 50 °C, then re-extracted with 2 -ml nitrile acetone and finally filtered through a syringe filter with pore size 0.45 μm. β-carotene content was measured by high performance liquid chromatography (HPLC), following the protocol modified by Haq et al. [38]. Briefly, the mobile phase consisted of methanol and water (9:1 *v*/*v*) with a flow rate of 0.8 mL/min. A UV detector was used at 472 nm under a column temperature of 25 °C.

Kinetics equations, widely used to describe changes in biological materials with several orders (0th–2nd), were evaluated (see in Equations (19)–(21)). Concentrations of β-carotene determined at each drying time interval were fitted to the 0th-, 0.5th-, 1st-, 1.5th-, and 2nd-order kinetics models. The best choice was chosen based on the highest *R^2^*, and lowest *RMSE* and *χ^2^*. The concentration ratio (*CR*) as a function of drying time for each temperature was expressed as follows [3,39]:

(19) $$\frac{d\left(CR\right)}{dt}=-k{\left(CR\right)}^{1-n}$$

Equation (21) was converted to logarithm form for different reaction orders:

(20) $$ln\left(CR\right)=-kt+b;\;(n=1)$$

(21) $${\left(CR\right)}^{1-n}=-kt+b;\;(n\;\neq \;1)$$

*CR* is *C/C*_0_ where *C* and *C*_0_ denote concentration of β-carotene at a specific and initial time, respectively, *t* is drying time (min), *k* is the rate constant of degradation and *b* is an equation constant. All constants in Equations (20) and (21) were determined by linear regression.

### 2.3. Experimental Setup and Model Validation

Validation experiments were conducted using a lab-scale tray dryer with chamber size 0.4 m × 0.7 m × 0.4 m. Ambient air was drawn through a heating box equipped with ten 1 kW finned heaters using a 1 hp blower (Mitsubishi Electric Automation Co., Ltd., Bangkok, Thailand). The drying air velocity was kept constant at 0.5 m s^−1^, controlled by an inverter (Model H-3200 Series, Haitec Transmission Equipment Co., Ltd., Guangzhou, China), and temperature was controlled by a PID controller (Model MAC-3D, Shimax Co., Ltd., Tokyo, Japan).

Carrot cubes were subjected to the drying process under various experimental scenarios. To investigate the kinetics of both volume shrinkage and degradation of β-carotene, carrot cubes were removed at different time intervals. All drying experiments for each condition were conducted in triplicate.

### 2.4. Synthetic Evaluation Index

Energy efficiency was maximized, while retaining dried product qualities in terms of shrinkage (*SH*) and β-carotene concentration (*BC*), by optimizing the drying condition. A synthetic evaluation index (SI) as a single process indicator was constructed and used to determine the most suitable drying mode. Relative parameters of SMER (*Y_1_*), volume shrinkage (*Y_2_*) and β-carotene concentration (*Y_3_*) were considered to calculate the SI. Model-predicted parameters for optimal drying condition with highest drying performance and product qualities were ranked in order of decreasing significance as—SMER, volume shrinkage (*SH*) and β-carotene concentration (*BC*). The weights of the ranked parameters were assigned as *λ_1_*, *λ_2_*, and *λ_3_* of 0.3, 0.2 and 0.1, respectively. Following [40], the SI value for each drying condition was calculated by Equation (22), with the most suitable drying condition chosen based on the highest SI value.

(22) $$S=\sum_{i=1}^{3}\lambda_{i}Y_{i}$$

(23) $$Y_{1}=\frac{SMER-SMER_{\min}}{SMER_{\max}-SMER_{\min}}$$

(24) $$Y_{2}=1-\frac{SH-SH_{\min}}{SH_{\max}-SH_{\min}}$$

(25) $$Y_{3}=\frac{BC-BC_{\min}}{BC_{\max}-BC_{\min}}$$

## 3. Results and Discussion

### 3.1. Description of Stepwise Drying Characteristics of Carrot Cubes Using a Combined Heat and Mass Transfer Model

#### 3.1.1. Model Validation for Moisture Content

**Figure 1:**
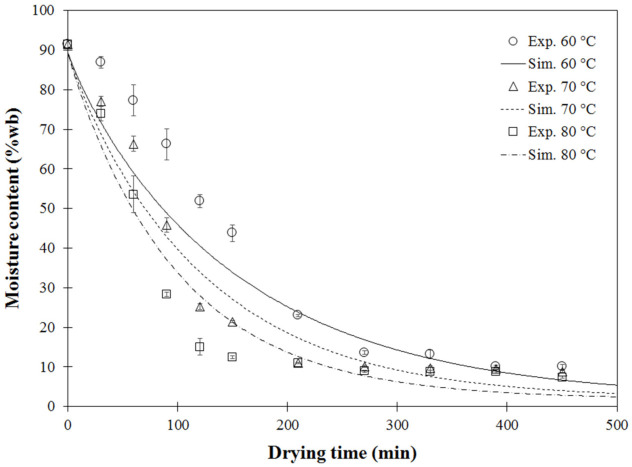
Comparison of experimental and model-predicted moisture content at different drying temperatures: Exp (experimental result), Sim (simulated result).

The combined heat and mass transfer model was first verified using the validation experiments of fixed drying air temperatures of 60 °C, 70 °C and 80 °C, as shown in Figure 1.

**Figure 2:**
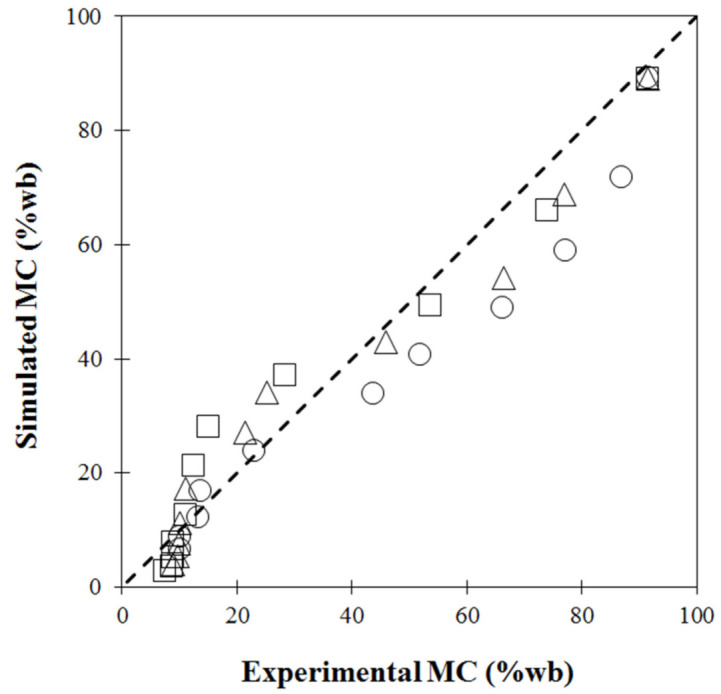
Validation plot of moisture content (%wb): circle (60 °C), triangle (70 °C) and square (80 °C).

For model validation, single-stage drying experiments were conducted with hot air temperatures of 60 °C, 70 °C and 80 °C. Simulated moisture contents as a function of drying time were compared with the experimental data, as shown in Figure 1. The simulated data showed a similar exponential trend as the experimentally measured data. This non-linear decrease was consistent with the common drying behavior of biological material [10,41]. Considerable agreement, confirmed by Figure 2, showed the model-predicted moisture content at each point close to the diagonal dashed line (y = x). However, discrepancy between the simulated and measured MC was observed, especially when MC was high or during the first drying period, due to the exclusion of large deformations or volume shrinkage caused by moisture removal [42] in the coupled heat and mass transfer equations used in the finite element model. Another explanation could be due to limitations of the simple model used. Empirical equations of volume shrinkage at different temperatures were introduced but not coupled into the heat-mass transfer model. At the initial stage, some mechanisms of temperature development, such as heat transfer resistance or thermal conductivity, could not be numerically captured [13].

#### 3.1.2. Effect of Stepwise Drying Mode on Drying Characteristic of Carrot Cubes

**Figure 3:**
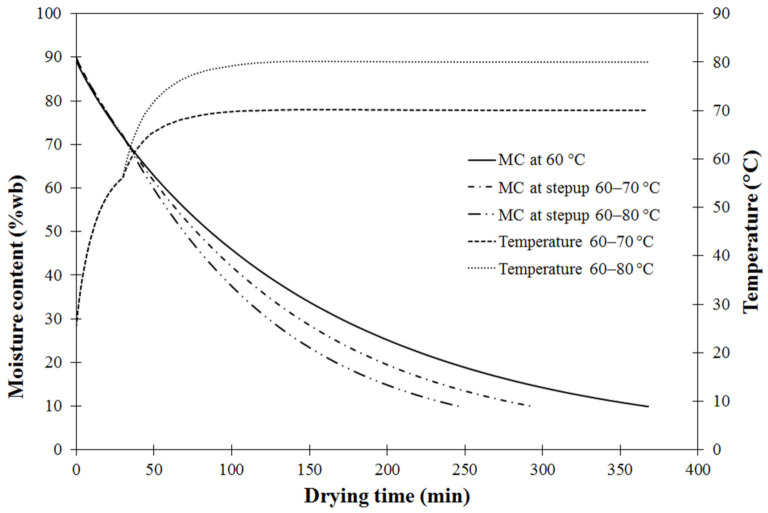
Comparison of simulated moisture content of carrot cubes at fixed 60 °C and two modes of step-up drying.

The verified coupled heat and mass transfer model was further used to describe drying behavior under two step-up modes of 60/70 °C and 60/80 °C operation compared to constant hot air temperature mode at 60 °C. As shown in Figure 3, for simulated moisture content of approximately 70% of initial MC, with drying air temperature increased to 70 °C and 80 °C at drying time of approximately 30 min, the carrot cube moisture content rapidly decreased after reaching the changing point, following [28]. This was explained by higher moisture diffusion speed in a falling-rate period when increasing drying temperature during the second stage. This increasing drying rate resulted in shorter drying time to reduce the moisture content to the desired level of 10%wb; reduced by 20% and 33% for step-up temperature of 60/70 °C and 60/80 °C, respectively. The model-predicted drying behavior obtained from these two step-up modes was consistent with results reported by [28], and Cuervo-Andrade and Hensel [43]. Using higher temperatures during the second drying stage when moisture content was low may affect the quality of dried products. Therefore, a suitable drying operation should be carefully chosen to maximize drying efficiency while also retaining final product qualities.

Table 2 shows the evolution of moisture content change with drying time under different drying modes. Moisture distribution is expressed by contour plots with colors ranging from red to blue indicating highest and lowest MC, respectively. The initial moisture content of the cube sample was assumed to be uniform for all operations, as seen in the red color at process onset. The changing point for stepping up the drying temperature was observed at around 30 min (see Figure 3), with moisture contours of all drying modes similar for the first 20 min. As drying progressed (after 40 min), differences in MC obtained from all tested drying conditions became evident. MC reduced faster at the 60/80 °C step-up drying mode, followed by the 60/70 °C step-up mode and the single drying stage at a fixed temperature of 60 °C. This observation was explained by higher thermal energy with increasing drying temperature during the second drying stage. After reaching the changing point, the driving force was boosted by increasing drying temperature in the second stage, resulting in higher moisture diffusion rate toward the surface, as shown by lower MC in the center compared to the single stage drying mode at the same drying time. Contour plots of moisture concentration for each drying time are presented in Table 2, showing uneven distribution inside the cube samples with MC highest in the center and continuously decreasing toward the cube surface. This was generally observed in the falling-rate drying period [11,31], confirming that the simple coupled heat and mass transfer model provided insights into the moisture migration path. High MC concentration observed in the center region supported the slowest heating zone, leading to low rate of moisture evaporation [4].

The verified model was subsequently used to calculate thermal energy consumption (Equation (13)), rate of water evaporation (*W_evap_*) and, consequently, specific moisture evaporation rate (SMER) of single-stage drying (constant 60 °C) and the two step-up drying modes (60/70 °C and 60/80 °C). Table 3 shows the predicted drying times to reduce moisture content of the cubes from the initial to the desired value of 10%wb under single-stage drying as 368 min, while the step-up modes of 60/70 °C and 60/80 °C provided shorter drying times by 20% and 33%, respectively. Total thermal energy consumption for single-stage drying gave the highest value, followed by two-stage drying of 60/70 °C and 60/80 °C, which reduced by 57% and 70%, respectively. Increased thermal energy due to higher drying temperature during the second stage was responsible for the negative effect on energy consumption saving. During the drying process, water evaporated at different rates under the various drying conditions tested. The highest value of *W_evap_* was recorded when the cube sample was dried under step-up mode with drying temperature of 60 °C used for the first stage and 80 °C for the second stage, while the lowest value was observed in single-stage drying.

The SMER value was also used as an index to evaluate drying mode performance, with a higher value inferring greater drying efficiency and reduced energy consumption. Results in Table 3 show that the step-up 60/70 °C mode had the highest drying performance with highest SMER of 0.5 × 10^−3^ kg/kWh, 66.67% higher than single-stage drying, while the step-up 60/80 °C mode gave a SMER value of 0.35 × 10^−3^ kg/kWh, higher by 16.67% compared to single-stage drying at 60 °C. Higher driving force for diffusing water with increasing drying temperature at the changing point could be a plausible explanation of this finding. This was consistent with the previous study of [28], reporting that the step-up drying process initially using drying temperature of 60 °C until reaching 70%wb, as the changing point, and 70 °C for the second stage toward the end of the process resulted in increased SMER by 50% when compared to the conventional drying at 60 °C. 

Results in Table 3 show that two-stage drying with 60 °C for the first stage and 70 °C for the second stage gave optimal drying performance with regard to energy saving. However, using a high drying temperature in the second stage where MC was low and its gradient was high may negatively affect both physical and chemical properties. Therefore, the best choice of drying process should consider drying performance and also the quality attributes of the final product.

### 3.2. Volume Shrinkage of Carrot Cubes

#### 3.2.1. Kinetics of Volume Shrinkage

**Figure 4:**
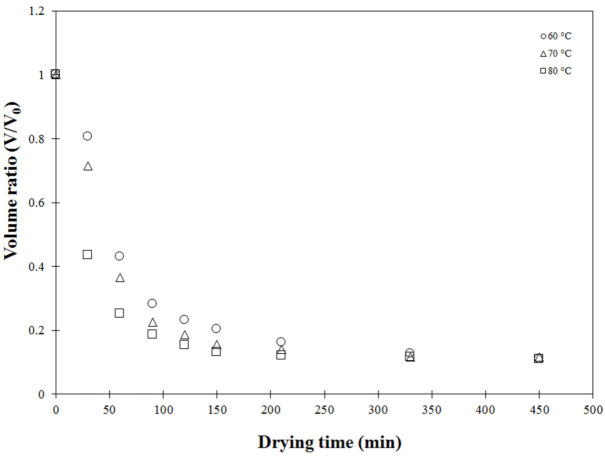
Experimental volume ratio (*V/V_0_*) as a function of drying time at different temperatures.

During drying, moisture or water losses in the cellular structure lead to change in shape and solid volume due to the dense and collapsed structure. This so-called solid shrinkage negatively affects the qualities of dried product, especially rehydration properties [37]. Figure 4 presents the measured volume ratio as a function of drying time at different operating temperatures. Under all drying temperatures tested, the volume ratio non-linearly decreased with drying time. Carrot is considered a semi-solid food as heterogeneous material consisting of 90% water [37]. In the convective hot air drying process, water is removed by evaporation, resulting in a pressure difference between the inner of the carrot cubes and the ambient pressure. This pressure unbalance generates contracting stresses resulting in depletion of the sample volume [37]. As a result, water removal positively influenced volume reduction, and shrinkage in terms of volume ratio decreased dramatically during the first drying stage with high drying rate. Change in material volume was also observed by Marques et al. [44], with slightly different volume ratios recorded when varying drying temperature, as shown in Figure 4. Rate of volume depletion during the first drying stage increased at higher drying temperature but was not obviously different in the later stages. This could be attributed to higher contracting stress caused by a higher rate of water evaporation when increasing drying temperature, consistent with the results reported by [6,45]. However, no difference in carrot shrinkage with a variation of drying temperature was found by [5].

The experimental shrinkage data in terms of volume ratio were fitted to various empirical models with different linear, quadratic, cubic and exponential functions. Table 4 presents statistical parameters in terms of *R^2^*, *χ^2^* and *RMSE* values to select the best shrinkage model for further use. 

Based on the highest *R^2^* and the lowest *χ^2^* and *RMSE*, the shrinkage model proposed by Ratti [35] was suitable to describe the change of carrot cube volume as a function of drying time, as expressed below:

(26) $$S=-0.061402+2.027857\cdot MC-5.10775\cdot MC^{2}+4.137980\cdot MC^{3}$$

(27) $$S=-0.091058+0.420872\cdot MC-1.10494\cdot MC^{2}+1.615213\cdot MC^{3}$$

(28) $$S=-0.011404+1.625343\cdot MC-4.19594\cdot MC^{2}+3.572144\cdot MC^{3}$$

Most research results showed a linear shrinkage profile versus moisture content for volume change during the drying of many materials such as carrot cubes [6] and carrot slices [45], while some results suggested a non-linear function [44,45,46]. 

The suitable shrinkage model was further used in the coupled heat and mass transfer model to investigate the effect of stepwise drying on volume depletion. The model was verified before serving as a tool for process analysis and optimization.

**Figure 5:**
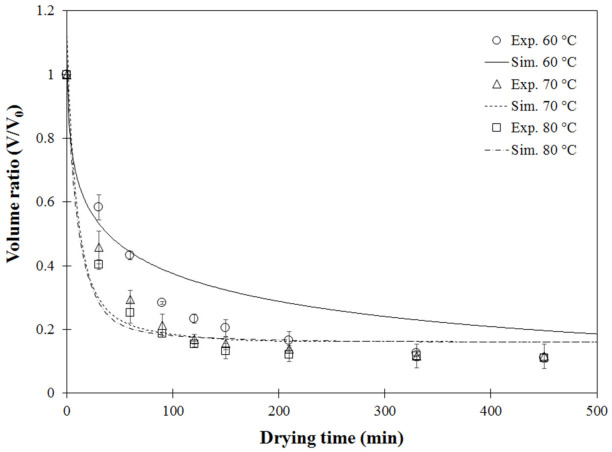
Validation results of shrinkage ratio of carrot cubes as a function of drying time at (circle) 60 °C, (triangle) 70 °C and (square) 80 °C.

**Figure 6:**
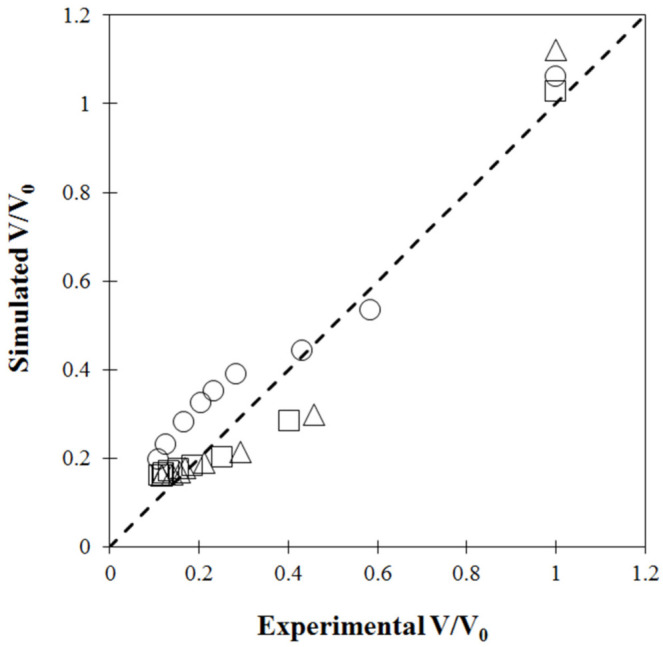
Validation plot of solid volume ratio (*V/V_0_*): circle (60 °C), triangle (70 °C) and square (80 °C).

Figure 5 compares experimental and model-predicted volume shrinkage as a function of drying time under different drying temperatures. Under the tested drying temperatures, incorporation of carrot shrinkage into the model provided acceptable fitting between the experimental and predicted data, except for results obtained from drying at 60 °C. The agreement was confirmed by most points lying close to the diagonal dashed line shown in Figure 6. Under this drying condition, a high discrepancy was found at the final drying stage where moisture content of dried carrot cubes was low. This could be attributed to the mild temperature used in the convective hot air dryer. This discrepancy was also observed by [13], using 56 °C to dry cocoa beans in a heat pump dryer.

Results obtained from the coupled heat and mass transfer model presented a non-linear trend similar to experimental observation. In the initial period of drying, water in carrot cubes was removed from the surface tissues under a high drying rate mechanism and the entire matrix was simultaneously dragged toward the center of the cubes at a rate strongly related to porosity and path of water diffusion following [4,11]. As drying progressed, moisture diffusion through the initial spaces in carrot cubes to the surface decreased. The samples shrunk slowly, due to decreasing mobility of the solid matrix, until reaching equilibrium. Under mild drying temperature of 60 °C, the solid matrix collapse mainly affected the volume change of carrot cubes instead of ideal shrinkage where the volume reduction is proportional to the mass loss [4,6]. In the final drying stage at which moisture content of the carrot samples was low, porosity only due to air-filled open pores was high. This could be a possible explanation of the overestimated shrinkage obtained by the model without this effect in our study [4].

#### 3.2.2. Effect of Stepwise Drying on Volume Shrinkage of Carrot Cubes

**Figure 7:**
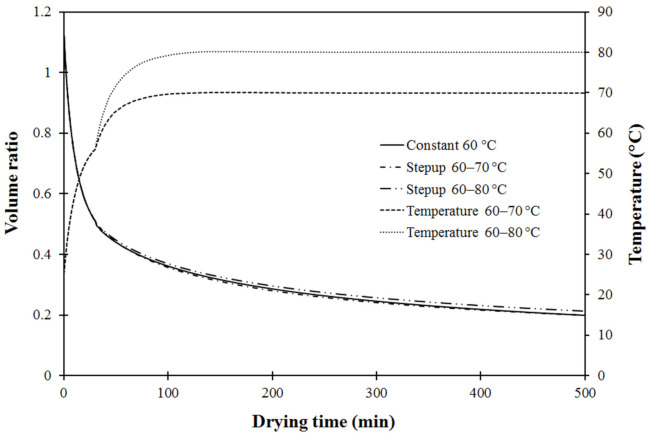
Comparison of simulated shrinkage ratio of carrot cubes at fixed 60 °C and two modes of step-up drying.

To investigate the effect of stepwise drying modes on volume shrinkage, three simulations using drying conditions of constant drying temperature of 60 °C and two step-up modes with 60 °C for the first stage followed by 70 °C and 80 °C for the second stage were performed using the previously verified model. Figure 7 shows the shrinkage of carrot cubes in terms of volume ratio at a certain drying time and the initial ratio (*V_t_/V_0_*) as a function of drying time, together with drying temperature profiles. 

During the last drying stage, increased drying temperature did not significantly affect volume shrinkage of the carrot cubes at a stepped-up temperature of 70 °C. This was consistent with the results obtained by Hatamipour and Mowla [47] who reported that volume shrinkage of carrot cubes dried in a spout-fluidized bed dryer was not significantly affected by air temperature. However, slight reduction in volume shrinkage was observed when using drying temperature of 80 °C for the second drying stage, as shown in Figure 7. Volume of the samples dramatically decreased in the first stage and continuously reduced during the second stage until the end of the process, with a model-predicted volume ratio of around 0.20 and 0.21 at stepped-up drying temperatures of 70 °C and 80 °C, respectively. This could be explained by the different mechanisms of volume change during drying process with variation of temperature. Instead of ideal shrinkage, when a reduction of solid volume directly relates to a loss of water, pores commonly appear depending on the moisture content and the drying conditions [6,48]. Drying temperature is an operating parameter that strongly impacts the porosity of the dried product. The negative effect of drying temperature on the volume shrinkage ratio during the second drying stage occurred because the drying rate at low moisture content was boosted by higher stepped-up temperatures, resulting in higher porosity of the dried cube samples [43]. During the drying process, simultaneous volume reduction and pore formation may have caused a continuous increase in porosity as drying progressed [49]. This observation concurred with the results reported by Senadeera et al. [50], stating that lower volume shrinkage of persimmon was observed at higher air temperatures ranging 45–65 °C. Wang et al. [49] also found that higher drying air temperature resulted in reduced shrinkage of banana slices.

### 3.3. Degradation Kinetics of β-Carotene in Carrot Cubes

#### 3.3.1. Modeling the Degradation Kinetics of β-carotene

β-carotene is a heat-sensitive bioactive compound in carrots. Kinetics models are now widely used to describe the dynamics of how constituent components degrade with operating time under thermal processes. The experimental data of β-carotene content profiles at different drying temperatures were fitted to various orders of kinetic equations. The most suitable model describing the degradation of β-carotene was chosen using the statistical parameters *R^2^* and *RMSE*, as shown in Table 5. 

According to the statistical parameters in Table 5, the 1st-, 1.5th- and 2nd-order kinetics equations gave *R^2^* value higher than 0.9, indicating good fit to the experimental data. However, when considering the error in terms of *RMSE*, the first-order kinetic model was the most suitable with lowest error value. Therefore, the coupled heat and mass transfer model associated with the first-order kinetics equation was used to describe the degradation of β-carotene content in carrot cubes during the drying process. Most studies on fruits and vegetables, especially carrot, report first-order reactions with respect to β-carotene concentration. Berruti et al. [51] studied the degradation kinetics of β-carotene during hot air drying at 70 °C and 80 °C using the first-order reaction. Results showed an effective kinetics model describing the change in β-carotene, which was highly influenced by the length of drying. Demiray and Tulek [3] and Goula and Adamopoulos [52] also used the first-order reaction to investigate β-carotene degradation in carrot slices during convective drying at temperatures ranging from 45 to 65 °C and 50 to 80 °C, respectively. The first-order kinetics model was effectively employed to describe the change in concentration of β-carotene during storage of dried carrot slices [11].

The degradation rate constants obtained from all drying experiments were linearly correlated with drying temperatures. The first-order degradation kinetics equation together with correlation of the rate constant (*k*) as a function of drying temperature is expressed below:

(29) $$CR=\exp\left(-kt\right)$$

and

(30) $$k=-0.0387+0.0008T\;(R^{2}=0.9937).$$

According to the correlation of the rate constant in Equation (30), drying temperature had a negative effect on the speed of β-carotene degradation; the higher the drying temperature, the faster the loss in β-carotene. This concurred with results reported by Berruti et al. [51], Demiray and Tulek [3] and Goula and Adamopoulos [52], showing that heat-sensitive β-carotene in carrot was degraded during the drying process, caused by degradation of natural antioxidants such as α-tocopherol [52,53].

#### 3.3.2. Model Validation for β-Carotene Content

**Figure 8:**
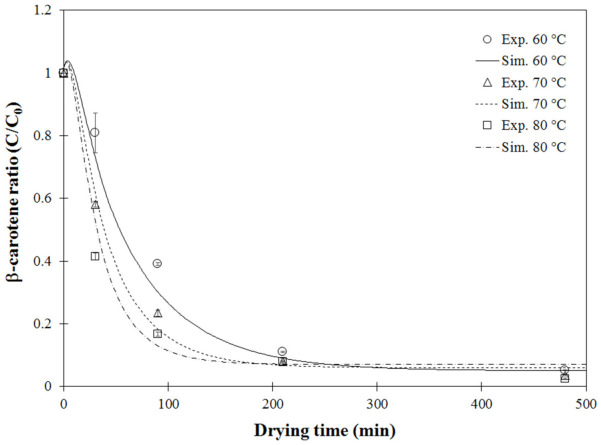
Validation results of β-carotene ratio of carrot cubes as a function of drying time at different drying temperatures.

Before investigating how the retention of β-carotene was affected by stepwise drying modes, the model-predicted ratios of β-carotene content as a function of drying time (min) were validated with the experimental β-carotene ratio of carrot cubes under drying temperatures of 60 °C, 70 °C and 80 °C, as shown in Figure 8. As drying progressed, the β-carotene content ratio, expressed as markers in Figure 8, decreased non-linearly from 57 to 59 mg/100 mg of dry matter to 2 to 3 mg/100 mg of dry matter. In the initial drying stage, β-carotene content reduced dramatically, with different degradation rates depending on drying temperature; the higher the temperature, the faster the degradation. This was due to the high heat sensibility of β-carotene when increasing drying temperature, concurring with many previous reports [3,11,51,54]. The simulated *C/C_0_* value as a function of drying time, expressed by continuous lines in Figure 8, also supported this observation.

**Figure 9:**
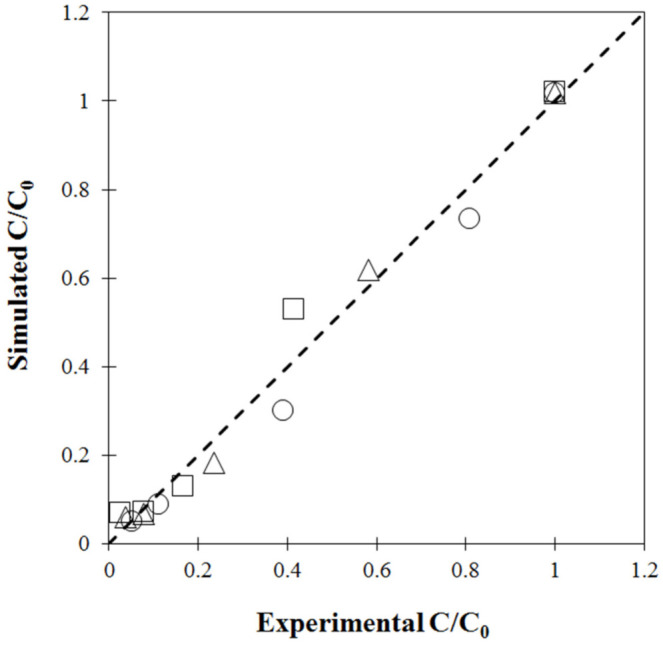
Validation of β-carotene ratio at different drying temperatures: circle (60 °C), triangle (70 °C) and square (80 °C).

The coupled heat and mass transfer model was verified by comparing the model-predicted *C/C_0_* values under different drying temperatures with those measured by HPLC. Results in Figure 9 show that most points are close to the diagonal dotted line, indicating a perfect fit and confirming that this model could be used for further investigation of stepwise drying.

#### 3.3.3. Effect of Stepwise Drying on β-Carotene Degradation

**Figure 10:**
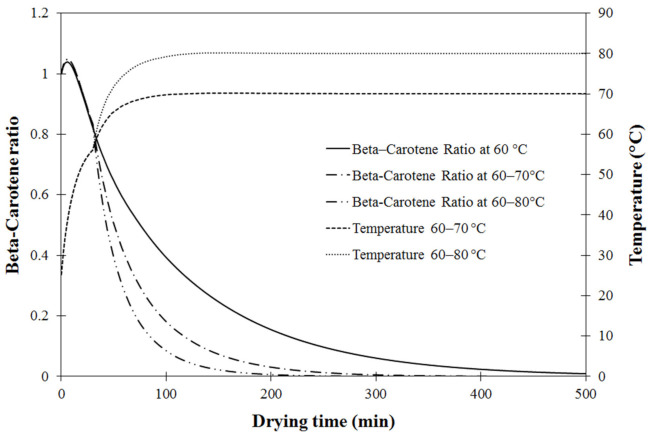
Comparison of simulated β-carotene ratio of carrot cubes at 60 °C and two modes of step-up drying.

According to the previous section concerning energy consumption as shown in Table 2, the step-up drying mode at 60 °C for the first stage and 70 °C for the second stage gave the best drying performance, with the highest SMER. However, levels of β-carotene in the dried carrot cubes were also considered when evaluating the drying efficiency because of commercial importance in the industry. The validated model was further used to investigate the effect of stepwise drying mode on retention of β-carotene in carrot cubes dried until reaching the desired moisture content of 10%wb, as shown in Figure 10.

As shown in Figure 10, the simulated β-carotene concentration ratio (*CR*) of carrot cubes dried under a fixed temperature of 60 °C, and at both step-up drying modes, decreased nonlinearly with drying time. This behavior indicated degradation of β-carotene commonly observed for bioactive compounds [3,54,55]. During the first drying stage at 60 °C, all *CR* curves were superimposed on each other, indicating identical profiles at the onset of the process until reaching the changing point of the step-up mode (~40 min). The β-carotene retention at drying time when moisture content reached the desired level of 10%wb was predicted for all tested drying modes. β-carotene ratios of carrot cubes dried under a fixed temperature of 60 °C, step-up mode of 60 °C to 70 °C and step-up mode of 60 °C to 80 °C were 0.054, 0.065 and 0.002, respectively corresponding to the remaining amounts of β-carotene at 3.11, 3.75 and 0.11 mg/100 g dry matter. After reaching the changing point in the second stage, as seen in Figure 10, the rate of β-carotene degradation increased. Demiray et al. [56] also reported that the rate of β-carotene degradation in dried tomatoes increased with increasing drying temperature from 60 to 100 °C, and the step-up mode using drying temperature of 70 °C for the first stage and 80 °C for the second stage highly impacted the degradation of β-carotene. This result concurred with Cuervo-Andrade and Hensel [43], who stated that stepwise drying reduced drying time and energy consumption but resulted in increased deterioration of quality attributes. Xanthopoulos et al. [10] found that product qualities, especially antioxidant properties, were significantly degraded at a drying temperature higher than 65 °C, while drying at lower than 55 °C retained higher quality but required longer drying time. Therefore, process optimization should be conducted based on drying performance that retained high or acceptable product qualities.

### 3.4. Synthetic Evaluation Index

The drying condition was optimized using the synthetic evaluation index (SI) with criteria of highest values of SMER and β-carotene content and lowest values of volume shrinkage. Table 3 shows the SI values predicted by the coupled heat and mass transfer model under various drying conditions, including single-stage drying at 60 °C, 70 °C and 80 °C and two step-up drying modes of 60/70 °C and 60/80 °C. A maximum SI value of 0.36 was obtained from the step-up 60/70 °C drying mode, followed by the single-stage drying mode at 70 °C with an SI value of 0.31, while the lowest SI value was found in the 60/80 °C drying. Consequently, the optimal drying condition for energy saving while retaining high qualities of solid volume shrinkage and retention of β-carotene was the step-up drying operation at 60 °C for the first stage and 70 °C for the second stage.

## 4. Conclusions

The simplified coupled heat and mass transfer model was used to describe the drying characteristics of carrot cubes in a convective hot air drying process by using a finite element approach. The model showed considerable agreement in drying characteristics, volume shrinkage and β-carotene content with experimental data obtained from single-stage drying runs with temperatures ranging from 60 °C to 80 °C. The verified model was subsequently used to investigate the effect of stepwise drying modes on drying characteristics, volume shrinkage and degradation of β-carotene. Drying under step-up modes of 60/70 °C and 60/80 °C gave shorter drying time by 20% and 33%, respectively, compared to single-stage drying with a constant temperature of 60 °C. This was explained by higher evaporation rate at increasing drying temperature during the second drying stage. Among the three drying conditions tested, the step-up 60/70 °C drying mode gave the highest SMER. This drying mode did not affect volume shrinkage but resulted in an extreme reduction in β-carotene after stepping up the drying temperature to 80 °C for the second stage. Based on the highest synthetic evaluation index, the step-up drying mode at 60 °C for the first stage and 70 °C for the second stage was the optimal operation, providing high energy efficiency while retaining good product qualities in terms of volume shrinkage and retention of β-carotene. However, this simplified model should be further improved by considering realistic volume shrinkage and other quality attributes.

## Figures and Tables

**Table 1:** Summary of empirical shrinkage models tested.

Reference	Equation	Equation
Lozano et al. [33]	$S=b_{1}\cdot MC+b_{2}$	(14)
Lozano et al. [34]	$S=b_{3}+b_{4}\cdot MC+b_{5}\cdot exp\left(\frac{b_{6}}{b_{7}+MC}\right)$	(15)
Ratti [35]	$S=b_{8}+b_{9}\cdot MC+b_{10}\cdot MC^{2}+b_{11}\cdot MC^{3}$	(16)
Vázquez et al. [36]	$S=b_{12}+b_{13}\cdot MC+b_{14}\cdot MC^{3/2}+b_{15}exp\left(b_{16}\cdot MC\right)$	(17)
Mayor and Sereno [37]	$S=b_{17}+b_{18}\cdot MR+b_{19}\cdot MR^{2}$	(18)

*S* is shrinkage coefficient, *b_i_* is numerical constants of empirical equations for shrinkage, *MC* is moisture content, g/g dry matter.

**Table 2:** Contour plots of simulated moisture content as a function of drying time.

Drying Time (min)	60 °C	60–70 °C	60–80 °C
0	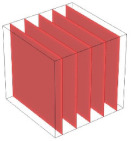	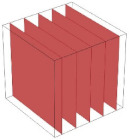	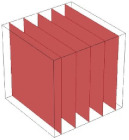
20	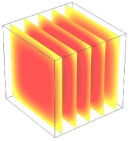	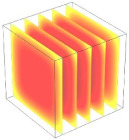	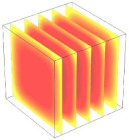
40	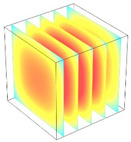	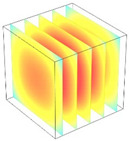	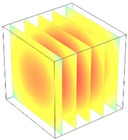
60	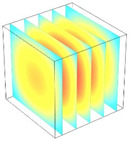	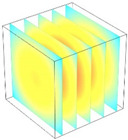	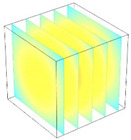
80	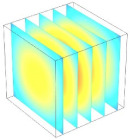	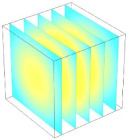	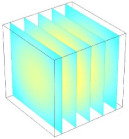
100	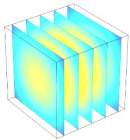	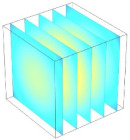	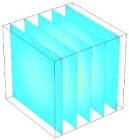

**Table 3:** Simulated results at the desired moisture content of 10% (wb).

Drying Mode	Drying Time (min)	Thermal Energy Consumption (MJ)	SMER (×10^−3^ kg/kWh)	Shrinkage Ratio	β-Carotene Content (mg/100 g DM)	SI
60 °C	368	74.01	0.30	0.3432	3.11	0.26
70 °C	288	76.20	0.23	0.1622	3.55	0.31
80 °C	238	76.97	0.19	0.1621	4.10	0.30
60–70 °C	294	22.29	0.50	0.2483	0.35	0.36
60–80 °C	247	32.05	0.35	0.1644	0.11	0.25

**Table 4:** Statistical results obtained from various empirical shrinkage models.

Model	Statistical Parameters	60 °C	70 °C	80 °C
Lozano [33]	*R^2^*	0.7301	0.8374	0.7909
	*χ^2^*	0.0314	0.0153	0.0192
	*RMSE*	0.1564	0.1093	0.1223
Lozano [34]	*R^2^*	0.9493 *	0.9773 *	0.9488 *
	*χ^2^*	0.0103	0.0104	0.0391
	*RMSE*	0.0678	0.0680	0.1318
Ratti [35]	*R^2^*	0.9971	0.9871	0.9963
	*χ^2^*	0.0005	0.0077	0.0359
	*RMSE*	0.0161	0.0656	0.1413
Vázquez [36]	*R^2^*	0.9352	0.9883 *	0.9885 *
	*χ^2^*	0.0132	0.0092	0.0417
	*RMSE*	0.0766	0.0641	0.1361
Mayor and Sereno [37]	*R^2^*	0.9500	0.9776	0.9500
	*χ^2^*	0.0068	0.0069	0.0262
	*RMSE*	0.0673	0.0680	0.1322

* Alias.

**Table 5:** Statistical parameters obtained from various orders of kinetic equation of β-carotene content.

Temperature	Statistical Parameters	0th	0.5th	1st	1.5th	2nd
60 °C	*R^2^*	0.7037	0.8076	0.9907	0.9709	0.9901
	*RMSE*	0.2010	0.2576	0.0362	0.3432	0.3254
70 °C	*R^2^*	0.5789	0.7230	0.9933	0.9712	0.9958
	*RMSE*	0.2349	0.2802	0.0297	0.3711	0.1583
80 °C	*R^2^*	0.4838	0.6675	0.9796	0.9924	0.9819
	*RMSE*	0.2568	0.3028	0.0511	0.3905	0.2107

## Data Availability

The data are available from the corresponding author.
